# Variation of hair cortisol in two herds of migratory caribou (*Rangifer tarandus*): implications for health monitoring

**DOI:** 10.1093/conphys/coad030

**Published:** 2023-05-22

**Authors:** F Rakic, X Fernandez-Aguilar, M Pruvot, D P Whiteside, G F Mastromonaco, L M Leclerc, N Jutha, S J Kutz

**Affiliations:** Department of Ecosystem and Public Health – Faculty of Veterinary Medicine, University of Calgary; 3280 Hospital Drive NW, Calgary, Alberta, Canada, T2N 4Z6; Department of Ecosystem and Public Health – Faculty of Veterinary Medicine, University of Calgary; 3280 Hospital Drive NW, Calgary, Alberta, Canada, T2N 4Z6; Department of Ecosystem and Public Health – Faculty of Veterinary Medicine, University of Calgary; 3280 Hospital Drive NW, Calgary, Alberta, Canada, T2N 4Z6; Department of Ecosystem and Public Health – Faculty of Veterinary Medicine, University of Calgary; 3280 Hospital Drive NW, Calgary, Alberta, Canada, T2N 4Z6; Reproductive Sciences Unit, Toronto Zoo, 361A Old Finch Avenue, Scarborough, Ontario, Canada, M1B 5K7; Department of Environment, Government of Nunavut, P.O. Box 377, Kugluktuk, Nunavut, Canada, X0B 0E0; Department of Environment and Natural Resources, Government of the Northwest Territories, 5112 52 st, Yellowknife, The Northwest Territories, Canada, X1A 2L9; Department of Ecosystem and Public Health – Faculty of Veterinary Medicine, University of Calgary; 3280 Hospital Drive NW, Calgary, Alberta, Canada, T2N 4Z6

**Keywords:** wildlife monitoring, wildlife health, stress, oestrid, mosquito, Arctic

## Abstract

Migratory caribou (*Rangifer tarandus* sspp.) is an ecotype of conservation concern that is experiencing increased cumulative stressors associated with rapid climate change and development in Arctic Canada. Increasingly, hair cortisol concentrations (HCCs) are being used to monitor seasonal hypothalamic–pituitary–adrenal axis activity of ungulate populations; yet, the effect of key covariates for caribou (sex, season, sampling source, body location) are largely unknown. The objectives of this research were 4-fold: first, we assessed the impact of body location (neck, rump) sampling sites on HCC; second, we assessed key covariates (sex, sampling method, season) impacting HCCs of caribou; third, we investigated inter-population (Dolphin and Union (DU), Bluenose-East (BNE)) and inter-annual differences in HCC and fourth, we examined the association between HCCs and indices of biting insect activity on the summer range (oestrid index, mosquito index). We examined hair from 407 DU and BNE caribou sampled by harvesters or during capture-collaring operations from 2012 to 2020. Linear mixed-effect models were used to assess the effect of body location on HCC and generalized least squares regression (GLS) models were used to examine the impacts of key covariates, year and herd and indices of biting insect harassment. HCC varied significantly by body location, year, herd and source of samples (harvester vs capture). HCC was higher in samples taken from the neck and in the DU herd compared with the BNE, decreased linearly over time and was higher in captured versus hunted animals (*P* < 0.05). There was no difference in HCC between sexes, and indices of biting insect harassment in the previous year were not significantly associated with HCC. This study identifies essential covariates impacting the HCC of caribou that must be accounted for in sampling, monitoring and data interpretation.

## Teaser

Hair cortisol concentration (HCC) can be a valuable indicator of stress in wildlife, but how to best implement this biomarker into existing caribou monitoring programs is unclear. Our results show that HCC is useful in understanding inter-annual and inter-population differences in caribou cortisol, which can inform future caribou management.

## Introduction

Migratory caribou (*Rangifer tarandus* sspp.) is a keystone ecotype in the Arctic that provides significant ecological, economic and cultural benefits to northern communities and ecosystems (Lyver, 2005; Côté *et al.,* 2010). Many migratory caribou populations have enigmatically and severely declined (by 70–90%) in the last decade (COSEWIC, 2016; COSEWIC, 2017; Campbell *et al.,* 2021). These declines may be associated with an increase in cumulative stressors, such as rising temperatures, increased frequency of extreme weather events and anthropogenic development in the Arctic environment (Vors and Boyce, 2009; Festa-Bianchet *et al.,* 2011; Gunn *et al.,* 2014; Fauchald *et al.,* 2017), among other stressors. Biting insect harassment is also a documented stressor of caribou that is anticipated to increase under current Arctic climate change scenarios of warmer temperatures, longer growing seasons and increasing precipitation (Witter *et al.,* 2012).

Stress responses in wildlife can be reflected in certain biomarkers of hypothalamic–pituitary–adrenal (HPA) axis activity, such as circulating glucocorticoids (GCs) (Baker *et al.,* 2013). GCs are involved in coordinating multiple physiological pathways in response to acute and chronic stress, including the mobilization of energy stores in mammals (Busch and Hayward, 2009). Prolonged and chronic elevation of GC concentrations can result in deleterious impacts such as reduced immunocompetence as well as decreased reproduction in mammals (Bonier *et al.,* 2009; Busch and Hayward, 2009). The dominant GC circulating in *Rangifer* is cortisol (Koren *et al.,* 2012), and this has previously been measured in the serum, feces and hair of *Rangifer* sspp*.* (Omsjoe *et al.,* 2009; Johnson *et al.,* 2010; Ashley *et al.,* 2011; Bondo *et al.,* 2019).

Hair cortisol concentration (HCC) is a promising biomarker that reflects chronic or cumulative stress experienced over weeks to months (Gormally *et al.,* 2020). Cortisol is believed to be incorporated into the hair shaft passively from the bloodstream during the anagen (active) hair growth phase, reflecting the HPA axis activity during that period (Russell *et al.,* 2012). Hair is a practical sample type to collect from caribou because it is a highly stable medium that can be easily transported and stored at room temperature (Felicetti *et al.,* 2003; Jaspers *et al.,* 2010), and it is already collected through community-based monitoring programs and during capture and collaring of caribou (Kutz *et al.,* 2013; Jutha *et al.,* 2022). Hair growth in *Rangifer*, although not precisely documented, occurs between June and October (Cuyler and Oritsland, 2002; Macbeth, 2013), when migratory caribou herds occupy their summer and fall ranges.

HCC as a biomarker of seasonally circulating cortisol has not been validated in *Rangifer* due to methodological constraints (Ashley *et al.,* 2011). However, studies in other free-ranging ungulates (*Oreamnos americanus, Ovibos moschatus*) that used sequential Adrenocorticotropic hormone (ACTH) injections during the hair growth period to simulate chronic HPA axis stimulation demonstrated higher HCC in the hair of experimentally treated animals compared with controls (Dulude-de Broin *et al.,* 2019; Di Francesco *et al.,* 2021). HCCs were previously used in *Rangifer* to investigate associations with body condition (Macbeth, 2013) and to monitor the effects of environmental disturbance (Ewacha *et al.,* 2017). However, there is a paucity of information examining key covariates (sex, season, sampling method, body location) of HCC that need to be considered to interpret HCC results in free-ranging *Rangifer.*

In this study, we sought to assess the use of hair cortisol as a biomonitoring tool in caribou and to examine the association between HCCs and indices of biting insect activity in the summer ranges of two migratory caribou herds: the Bluenose-East (BNE) (*Rangifer tarandus groenlandicus*) and the Dolphin and Union (DU) (*R.t. groenlandicus x pearyi)* herds. We asked four questions: (1) Is there a significant difference between HCCs of neck and rump hair sampled from the same individual in caribou? (2) What factors associated with the opportunistic sampling of caribou (sex, season, body location, sampling method) are significant covariates of HCC? (3) Does HCC exhibit annual trends and inter-population differences? And (4) is hair cortisol positively associated with the intensity of biting insect harassment?

## Methods

### Study area and sample collection

**Figure 1:**
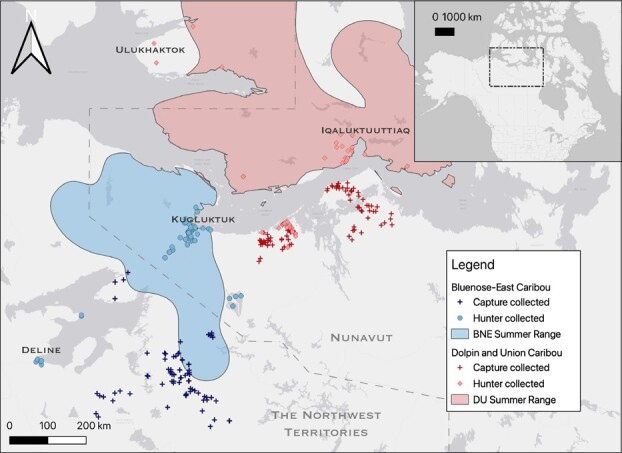
Map of BNE (blue) and DU (red) sampling locations, as collected by hunters (circles) and on captures (crosses) between 2012 and 2020. Summer ranges were delineated from CircumArctic *Rangifer* Monitoring and Assessment Network database (Russell *et al.,* 2013).

The study populations were the BNE herd, which ranges east of Great Bear Lake and west of Kugluktuk, Northwest Territories (NWT), and Nunavut (NU), and the DU herd, which ranges on Victoria Island and mainland NU and NWT (Committee on the Status of Endangered Wildlife in Canada (COSEWIC, 2016) (Fig. 1). The BNE and DU herds have been federally assessed as threatened and endangered, respectively (COSEWIC, 2016, 2017). Hair samples from both herds were collected and archived by two opportunistic sampling methods. The first method was by subsistence hunters through community-based monitoring programs from 2012 to 2021 from the communities of Délįnę, Norman Wells and Ulukhaktok of the NWT and from Kugluktuk and Iqaluktuuttiaq, NU. The second method was by convenience sampling during live capture and collaring activities performed by the Governments of the NWT and NU. The duration of caribou capture is ~15 min, including hair sampling (Cattet, 2018). Paired neck (lateral side) and rump samples from 52 individual caribou were collected in 2019 during a DU caribou harvest (*n* = 18) and in 2021 during a capture/collaring project (*n* = 34).

Hunted samples were collected year-round, but predominantly during common harvesting periods: the spring/summer for the BNE and fall/spring for the DU. Hair samples from hunted animals were collected almost exclusively from the neck and/or rump as requested in the collection protocol. Samples from captured caribou were collected during March for the BNE herd and April for the DU herd. Hair samples collected during capture from the BNE were taken from various body locations, including the neck, rump and shoulder, whereas DU capture samples were taken consistently from the neck and rump. Samples collected from capture were generally of plucked hair that contained a hair bulb and follicle. Hair samples collected by hunters were predominantly submitted on the hide, and hair was shaved as close to the skin as possible using a stainless steel razor. A sufficient mass of hair for analysis was collected and submitted from 201 BNE caribou (84 hunted, 117 captured) and 206 DU caribou (116 hunted and 90 captured). Samples from the BNE herd were approximately equal between sexes (F = 106, M = 97), however, for the DU herd, predominantly female caribou were sampled (F = 171, M = 35).

### Sample processing and laboratory analysis

All hair cortisol quantification was performed by the Toronto Zoo’s Endocrinology Laboratory (Scarborough, Canada). Hair decontamination, preparation and steroid extraction were completed as described by Mastromonaco *et al.* (2014) and used by Di Francesco *et al.* (2021). Briefly, hair samples were examined under a dissecting microscope at 10× power, and hair follicles, bulbs and undercoat, if present, were manually removed such that solely the guard hair remained. Samples were washed by immersing them in 700-ml plastic containers filled with distilled water, rubbed by a gloved hand for 2 min and then dried in a paper towel. Hair was then placed in 20-ml glass vials, vortexed with 15 ml of distilled water for 10 s, then soaked for 5 min. The liquid was removed, and another 15 ml of distilled water was added, vortexed for 10 s and then immediately removed. Finally, 15 ml of 100% methanol was added, vortexed for 10 s and removed immediately. Completely washed hair was dried in a paper towel and then stored in paper envelopes at room temperature.

Washed and dried hair was cut into 5-mm pieces and weighed into 7-ml scintillation vials. Fifty milligrams of hair were extracted with 100% methanol, for a ratio of 0.01 g of hair/ml of methanol, on a rotator plate (MBI Lab Equipment orbital shaker, 100 rpm) at room temperature for 24 h. Samples were centrifuged for 5 min (at 2400*g*), and the supernatant was pipetted into new glass 7-ml vials. Supernatants (hair extracts) were then stored at −20°C. For cortisol quantification, stored hair extracts were brought to room temperature, and 1500 μl of hair extract was evaporated in a fume hood; the dried extract was then reconstituted using 150 μl of enzyme immune-buffer forming a 10× concentration. Cortisol was quantified using EIAs previously described by Majchrzak *et al.* (2015) and Kummrow *et al.* (2011) and used by Di Francesco *et al.* (2021), and all samples and standards were run in duplicate.

### Data processing

**Figure 2:**
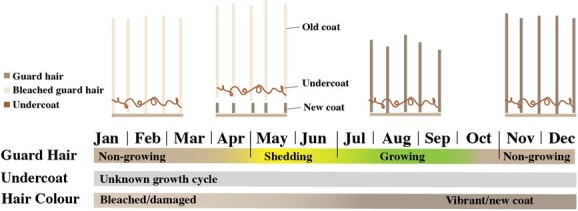
Summary of annual caribou guard hair growth cycle (Cuyler and Oritsland, 2002; Macbeth, 2013). Guard hair is primarily shed (May/June) and grown (June–September) once annually. A fine woollen undercoat is also present in caribou pelage (Ewacha *et al.,* 2017); however, the growth cycle is unknown. Caribou guard hair tends to become bleached and damaged as the hair ages over winter (Macbeth, 2013, Rakic personal obs.).

Depending on the date of hair collection, hair was either representative of the current or previous year of guard hair growth (Fig. 2), requiring hair growth year to be individually classified. Active guard hair growth is understood to occur between June and September in *Rangifer* (Cuyler and Oritsland, 2002; Macbeth, 2013). Thus, hair collected between October and December was classified as current year hair growth and hair collected between January and June was classified as previous year growth. Hair collected between July and the end of August (*n* = 33), during the period of active growth, was classified based on hair length and colour (*i.e.* long, white, damaged hair signified previous growth, and short, brown hair: current year growth).

Sample and demographic information such as date of sample collection, location and sex of animal were obtained from hunter forms and datasheets completed by government wildlife handling staff. Ordered month categories, beginning in September when hair was considered new growth, were created for data analyses. These categories were: 1 = September/October, 2 = November/December, 3 = January/February, 4 = March/April, 5 = May/June and 6 = July/August. Month categories were treated as continuous to test the effect of seasonal changes in hair on HCC. Year was assigned based on hair growth year, not the year of sample collection or analysis. Age was determined based on tooth eruption patterns (calf, sub-adult, or adult) (Miller, 1972) or as reported on hunter and/or capture forms. Only adults (>3 years old) and sub-adults (1–3 years old) were included in statistical analyses; 8 calves (<1 year old) were removed (6 DU, 2 BNE). Furthermore, solely animals of known sex were included, removing a further 10 animals (all BNE caribou), resulting in a sample size of 407 animals for data analysis.

Oestrid and mosquito activity indices for the BNE and DU summer ranges were determined following the guide provided by Witter *et al.* (2014) and were obtained from the CircumArctic *Rangifer* Monitoring and Assessment Network MERRA database (Russell *et al.,* 2013). Indices were restricted to the period of biting insect activity from June 15 to September 1 (Witter *et al.,* 2014), and the average activity for that period was calculated. If a caribou was harvested between June and September, then the mean insect harassment index was calculated up to the date of harvest for that individual animal (*i.e.* an animal harvested on August 18 would have average harassment from June 15 to August 18). HCCs were log-transformed to satisfy assumptions of linear regression. Samples below the limit of hair cortisol quantification (*n* = 19), reported as <1.00, were treated as low values and assigned a value of 0.5 pg/mg (Vikøren *et al.,* 2011).

### Statistical analysis

All analyses were completed in R version 3.6.0 (R Core Team, 2020). To determine the effects of body location (rump or neck), we used a paired hair dataset from 34 captured adult female DU caribou and 18 hunted (5 male, 13 female) DU caribou, totaling 52 pairs (*n* = 104 total). We compared HCCs in hair from the rump and neck by first using a Wilcoxon signed-rank test to test for differences in rank distribution. Second, an F-test was used to compare possible differences in variance between body locations. Third, correlations between neck and rump hair concentrations were measured using Pearson correlations. Last, the differences by body location (rump or neck) were assessed using a linear mixed-effects model (lme4 R package (Bates *et al.,* 2015). Animal ID was fit as a random effect to account for repeated sampling of the same individual (Paterson and Lello, 2003), and body location (neck/rump) was a fixed effect explaining the log HCC in 52 animals. In a sensitivity analysis, outlier values were detected (*n* = 2) using a Rosner test using the EnvStats package (Millard, 2013), and all statistical analyses were repeated in the absence of outlier values.

Using the full dataset of all archived DU and BNE hair (*n* = 407 individuals), we assessed the association of multiple covariates with HCC. Models were fit with seven fixed effects: sex (male/female), herd (BNE/DU), month category (1–6), Oestrid index (OI), mosquito index (MI), body location (neck, rump, shoulder or unknown), sampling method (hunted/captured), and year (2012–2020). Ordered month categories (1–6) were treated as continuous to evaluate the effects of hair aging and seasonal change. Rump values were used for the DU caribou (*n* = 54), in which both neck and rump values were available. Mixed-effect linear regression was fit using the lme() function. However, after log-transformation, box-cox transformation and weighted regression, non-normality of residuals and heteroskedasticity of best fit models were detected. To overcome these barriers to analysis, generalized least squares regression (GLS), which is more robust to violations of standard regression assumptions (Beale *et al.,* 2010), was done using the gls() function from “nmle” package. Based on multiple top models including different covariates and being within 2 Akaike Information Criterion (AIC_c_) of one another, model averaging was used to generate covariate estimates, confidence intervals and statistical significance. The global model was fit, and all possible combinations of variables were fitted in separate models using ‘MuMIN’ package (Bartoń, 2020). The top models that were within 2 AIC_c_ of one another were averaged using the model.avg() function, and averaged effect sizes of included fixed effects were extracted. Lastly, collinearity was assessed by examining variance inflation factors and using a cut-off value of 5 (James *et al.,* 2013).

## Results

Eleven immune-assay plates were run between July 2019 and June 2021. Inter-assay coefficients of variability (CVs) were 17.1% (25% binding) and 20.1% (60% binding), and intra-assay CV was 8.5%. Hair cortisol ranged from <1.00 pg/mg to 51.89 pg/mg (Table 1).

**Table 1:** Sample size (*n*), median and range (min, max) of guard HCC (pg/mg) from migratory caribou from the BNE and DU herds from 2012 to 2020

		2012	2014	2015	2016	2017	2018	2019	2020
BNE	***n***	**3**	**21**	**17**	**65**	**27**	**23**	**29**	—
	med	2.53	2.21	3.15	3.50	2.29	2.28	2.16	—
	min	1.52	1.06	1.17	0.50	0.50	0.50	0.50	—
	max	3.28	6.36	8.91	51.89	15.21	6.12	8.06	—
DU	*n*	**—**	**—**	**—**	**—**	**90**	**40**	**22**	**54**
	med	**—**	**—**	**—**	**—**	8.84	5.63	2.96	4.32
	min	**—**	**—**	**—**	**—**	2.91	1.54	0.50	1.41
	max	**—**	**—**	**—**	**—**	24.76	12.01	6.82	13.29

### Body location and hair cortisol concentration

**Figure 3:**
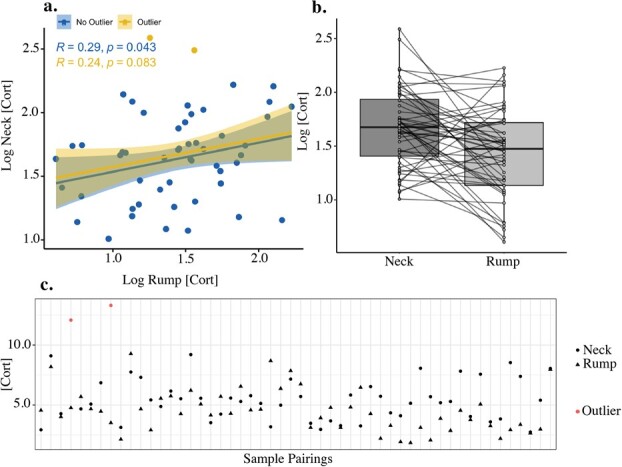
Comparison of log cortisol concentration (pg/mg) of caribou hair between paired neck and rump samples of 52 unique DU caribou from 2021. (a) Scatter plot comparing log neck and rump concentrations, with Pearson correlation (line), associated 95% confidence interval (shading), Pearson correlation (R) and associated *P*-value of outlier (yellow) and outlier-removed (blue) data. (b) Boxplot comparing log median neck and rump concentrations, lines connect data points from the same individual. (c) Scatter plot showing untransformed hair cortisol (pg/mg) between neck (circle) and rump (triangle) pairings and outliers (red).

Cortisol concentrations were not significantly influenced by body locations using a Pearson (r = 0.24, *P* = 0.083) correlation (Fig. 3). However, on removal of two outlier values, cortisol concentrations between body locations were weakly correlated with one another (r = 0.29, *P* = 0.0043). Neck hair sample variance (4.88 pg/mg) tended to be greater than that of the rump hair (3.23 pg/mg), but these variances were not statistically different (F = 0.824, *P* = 0.492). Median rank values significantly differed and were higher in neck samples in a paired comparison (Wilcoxon *P* = 0.004). Results from linear model analyses (Supplementary Materials, Table S1, Table S2) demonstrate that body location was a significant covariate predicting HCC, and concentrations were higher in the neck hair (*P* < 0.001). All linear models adhered to the assumptions of linear regression analysis (Supplementary Materials, Figure S1).

### HCC in migratory caribou

Of 256 total possible combinations of models to predict caribou HCC, the four top models were within 2 AIC_c_ of one another (Supplementary Materials, Table S3). Five parameters—year, herd, month, body location and sampling method—were significant predictors of HCC. Non-significant covariates included the indices of oestrid and mosquito activity and sex. Interaction terms, although included in the set of models, were not present within top fit models from which averages were derived.

**Figure 4:**
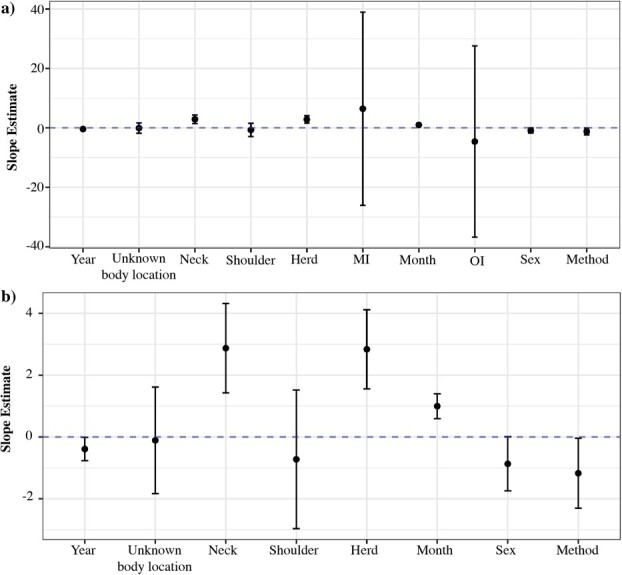
Forest plot of covariates (*x* axis) predicting HCC in caribou with the associated GLS regression slope estimate (*y* axis). The 95% confidence intervals are denoted. Estimates were averaged from four top models (ΔAIC_c_ < 2). Covariates included are year (2012–2020), unknown, neck and shoulder body locations (rump reference), herd (BNE reference), MI (mosquito index), OI (oestrid index), month categories (1–6), sex (female reference) and sampling method (capture reference). a) with all covariates, b) MI and OI are removed to visualize other covariates.

Five covariates whose 95% confidence intervals did not include zero were significant: year (2012–2020), herd (DU/BNE), month category (1–6), body location (neck) and sampling method. Sex (*P* = 0.052) was borderline significant, and males were lower in HCC compared with females; however, when outliers were removed, sex was non-significant (*P* = 0.17) (Supplementary Materials, Table S5). The herd was a significant covariate in the four averaged models (*P* < 0.001), with the DU herd HCC being consistently higher compared with the BNE. Month category followed a positive linear trend in both herds such that HCC concentrations were increasing from September to October with a peak in May to June (Fig. 4) and was included in all top models. Year was a significant predictor of HCC (*P* = 0.04) and cortisol concentrations decreased linearly for both herds—2012–2019 for the BNE and 2017–2020 for the DU—and was included in three of the top models. The sampling method was included in all top models, and HCC was higher in captured animals (*P* = 0.04). Lastly, the mosquito and oestrid indices were included in all top models, but both estimates include zero in the 95% confidence interval and were therefore non-significant (Supplementary Materials, Table S4, Table S5).

## Discussion

For HCC to be a useful biomarker of stress, the influence of covariates must be considered in the study design and results interpretation. We demonstrated that, for caribou, the key covariates of body location, season and sampling method (captures vs harvested) influenced HCC, whereas sex seemed not to, but should still be considered in future analyses. Accounting for the covariates, we detected significant inter-herd differences, as well as decreasing annual values in HCC that are of wildlife management relevance. We did not, however, detect an influence of oestrid flies or mosquitoes. Values of HCC ranged within and above wild (Ewacha *et al.,* 2017) and captive (Ashley *et al.,* 2011; Carlsson *et al.,* 2016) values previously reported in *Rangifer* sspp. Wild muskoxen (*Ovibos moschatus*), sympatric with the DU caribou herd, have a similarly wide range of HCC values (Di Francesco *et al.,* 2017).

### Differences between body locations

Health monitoring programs for caribou have collected hair from multiple different body locations (neck, rump and shoulder); however, HCC may vary among body locations, depending on species and study (Macbeth *et al.,* 2010; Ashley *et al.,* 2011; Macbeth *et al.,* 2012; Carlitz *et al.,* 2013; Carlsson *et al.,* 2016; Schell *et al.,* 2017; Acker *et al.,* 2018). We detected significant differences in HCC between neck and rump locations in caribou, and body location was a predictor in paired HCC models.

Differences in HCC between body locations are attributed to various factors, such as hair colour (Ashley *et al.,* 2011; Acker *et al.,* 2018), grooming behaviour (Acker *et al.,* 2018), local cortisol production (Macbeth *et al.,* 2010) and heterogenous molting (Macbeth *et al.,* 2010; Heimbürge *et al.,* 2020). Differences in molting (hair growth covering different periods) and hair length (differences in growth speed or time frame) are the most likely cause for the differences in HCC between body locations (Macbeth, 2013). Although not specifically described for caribou, using moose growth patterns as a proxy, coat replacement begins in the ventral and cranial areas and proceeds dorsally and caudally, and the margins of the rump are generally the last area to be shed (Samuel *et al.,* 1986). In caribou, neck hair is highly variable, with males growing a long mane or beard. We observed a lower variance and lack of outlier values for the rump hair compared with the neck. A similar pattern of increased variability of neck hair was observed in muskoxen (Di Francesco *et al.,* 2021). It follows that standardized sampling in *Rangifer* should target rump hair, with the caveat that local inflammation induced by oestrid larvae, which occur below the skin on the back and rump, may be a source of local cortisol production and hair contamination.

### Sampling method differences

Caribou hair samples are derived from two primary opportunistic sampling methods (hunts and captures), and we found that hair from captured animals had significantly higher HCC values. The mechanistic explanation for these differences in biomarker outcome depending on sampling methodology is unclear. A possible conclusion is that all captured samples were plucked and included the hair follicle, which can increase HCC if not removed (Sergiel *et al.,* 2020), and hunted samples were mostly shaved (lacking a follicle). These follicles are manually removed by hand, and it is likely that not all are removed before hormone extraction, possibly biasing results. Furthermore, there may be a potential sub-population sampling biased between helicopter-based capture and land-based hunting. Acknowledging these differences between sampling methods, the sampling method must be accounted for in future HCC analyses and interpretations when combining samples from multiple sampling methods.

### Sex differences

Varied results are reported concerning sex differences of HCCs in ungulates because sex-unique physiology or behaviours during active hair growth may be species-specific. Studies have reported no difference in HCC between sexes in red deer (*Cervus elaphus*) (Caslini *et al.,* 2016) and white-tailed deer (*Odocoileus virginianus*) (Potratz *et al.,* 2019), higher HCCs in male moose (*Alces alces*) (Madslien *et al.,* 2020) and muskoxen (*O. moschatus*) (Di Francesco *et al.,* 2017) and higher HCCs in female Rocky Mountain goats (*Oreamnos americanus*) (Dulude-de Broin *et al.,* 2019) and alpine ibex (*Capra ibex*) (Prandi *et al.,* 2018). In the present study, female caribou tended to have higher HCC compared with males, and these results were not statistically significant when outlier values were removed. Physiologically, differences in body condition, energy mobilization and body size can alter HCCs (Heimbürge *et al.,* 2019), which may explain a potential sex difference; however, this study did not have access to complete body condition nor body mass information to explore these relationships. Nevertheless, sex may still present a significant confounding factor in HCC analyses and interpretations that should be accounted for.

### Seasonal variation

**Figure 5:**
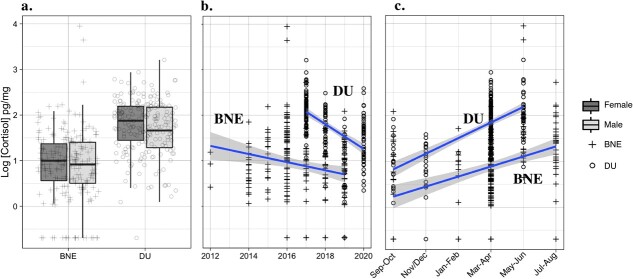
Combined log hair cortisol values (pg/mg) for all animals included in statistical modelling by (a) herd and sex, (b) year (circles = DU herd, crosses = BNE herd) and (c) season. Blue lines correspond to regression lines and grey shading 95% confidence intervals of model fit. Regression lines separated by herd (BNE and DU). Thick horizontal lines of boxplots correspond to medians (dark grey = female, light grey = male).

The month of hair collection was a significant predictor of HCC in caribou, and these values tended to increase from September to October and peak in May to June (Fig. 5). Hair cortisol values derived in this study are believed to reflect what is present in the hair shaft after a wash procedure. This ‘internal’ cortisol would reflect the circulating free cortisol as well as local cortisol synthesis taking place during the active hair shaft growth period (Ito *et al.,* 2005; Keckeis *et al.,* 2012; Russell *et al.,* 2012), which is June–September for caribou (Cuyler and Oritsland, 2002). We found that HCC levels in the herds increased in the hair shaft during the non-growing quiescent phase (October–June), suggesting cortisol is variably incorporated into the hair beyond the period of active growth. Similar findings in grizzly bears (*Ursus arctos*) showed a significant increase in HCC beyond the hair growth period (Cattet *et al.,* 2014), and seasonal changes in HCC occur in boreal snowshoe hares (*Lepus americanus*) (Lavergne *et al.,* 2020), grey wolves (*Canis lupus*) (Pereira *et al.,* 2022) and Vancouver Island marmots (*Marmota vancouverensis*) (Acker *et al.,* 2018). Continuous hair growth and local cortisol production and incorporation into the hair shaft are possible explanations for this phenomenon.

The chronology of caribou hair growth is not well described; however, it is thought that most guard hairs grow through the summer and that the density of pelage is stable year-round (Cuyler and Oritsland, 2002). Although guard hairs seem to be stable in density, there is a possibility that a select percentage of hair is shed and replaced continuously. In muskoxen, 20% of guard hairs are continuously replaced on the rump year-round (Flood *et al.,* 1989). Our finding of peak HCC in spring is consistent with seasonal-specific stressors in caribou; a peak of HCC in May/June corresponds to spring migration to calving grounds after a long winter (Taillon *et al.,* 2012). Caribou also have a fine wool undercoat that is present throughout the year (Cuyler and Oritsland, 2002). Although this may have a different HCC than guard hairs, it is manually removed before cortisol extraction.

Incorporation of locally produced cortisol into the hair shaft may also be a reason for the increased HCC beyond the hair growth period. Sweat from apocrine glands contains cortisol (Russell *et al.,* 2014), and any cortisol-containing fluid can diffuse into the hair shaft (Otten *et al.,* 2020). The capacity of external substances to diffuse into the hair shaft may also be related to hair integrity and age. Indeed, damaged or distal hair tends to be more susceptible to external cortisol penetration from sweat or sebum (Heimbürge *et al.,* 2020). Conversely, it has also been hypothesized that hair cortisol may be capable of washout in response to grooming or environmental exposure (Acker *et al.,* 2018). Caribou pelage undergoes a marked change during the winter from grey/brown in the fall to white in winter, alluding to a change in hair integrity. This may facilitate the incorporation of locally produced cortisol into the hair shaft. Significant variation of HCC beyond the hair growth period demonstrates that season is a key covariate to consider when interpreting health monitoring data derived from multiple months.

### Herd differences

The DU caribou population had significantly higher HCCs compared with the BNE barren-ground caribou herd. The DU herd is a distinct ecotype and subspecies of barren-ground caribou, as defined by morphological, behavioural and genetic characteristics (COSEWIC, 2011). Ecologically, DU caribou range on Victoria Island and seasonally migrate to the mainland across sea ice (GNWT, 2018). On the other hand, the BNE herd ranges on the mainland and seasonally migrates between the tree line and the tundra (COSEWIC, 2016). Prominent ecological, behavioural and anthropogenic pressure differences between these herds may be reflected by differences in baseline HCC. Moreover, ungulate populations inhabiting differing local environments exhibit differing average cortisol concentrations (Caslini *et al.,* 2016). Morphologically, DU caribou are considerably smaller in size and have a lighter white or grey pelage compared with the BNE. Numerous studies have reported hair colour as a significant predictor of HCC (Burnett *et al.,* 2014; Heimbürge *et al.,* 2019), and in some cases, elevated values in lighter coloured hair (Bennett and Hayssen, 2010; Bowland *et al.,* 2020). Coat colour differences between caribou ecotypes may present a confounding factor in comparing HCCs between populations and may be contributing to the differences reported by this study.

### Biting insect harassment

We did not detect a significant association between indices of biting harassment and HCC in the two study groups of caribou. This was unexpected given the behavioural impacts of biting insect harassment on *Rangifer sspp*. (Toupin *et al.,* 1996; Weladji *et al.,* 2003) and the suggested capacity of HCC to reflect impacts of disturbances in caribou (Ewacha *et al.,* 2017). We were limited by solely having access to and considering mosquito or oestrid presence, when black flies (*Simuliidae spp.)* and horse flies (*Tabanidae spp.)* may also be significant stressors (Toupin *et al.,* 1996). Statistically, these indices are proxies derived from weather data and only document the total hours of moderate to high insect activity (Witter *et al.,* 2012). Furthermore, these indices are herd-level measurements, and high inter-individual variability in HCC may have prevented the detection of a population-level association. Biologically, biting insect harassment as a potential stressor is endemic to these populations, and migratory caribou may have an adaptive tolerance strategy resulting in a limited HPA axis response (Cizauskas *et al.,* 2015). A tolerance strategy hypothesis is proposed to explain the lack of association between HCC and helminth parasite infection (Carlsson *et al.,* 2016; Trevisan *et al.,* 2017; Di Francesco *et al.,* 2022) and may be extended to insect harassment.

### Annual trends

We detected significant differences of annual HCC in migratory caribou. HCC differed between years (model results) and linearly declined in both herds (Fig. 5). During this same time frame, both populations greatly declined in size; the BNE herd declined by 84% from 2012 to 2021 (Adamczewski *et al.,* 2017; Boulanger *et al.,* 2019), and the DU herd declined by 90% from 1997 to 2020 (COSEWIC, 2017; Campbell *et al.,* 2021). However, HCCs decreased in both herds from 2012 to 2020. Density-dependent effects are documented in *Rangifer* populations (Solberg *et al.,* 2001), and density, alongside environmental factors, influences the dynamics of these populations (Tews *et al.,* 2007). Thus, caribou decline and subsequent relaxation of density-dependent constraints may be associated with a decrease in biomarkers of HPA axis activity. This hypothesis is partially corroborated by findings in woodland caribou (*R.t. caribou*) (Ewacha *et al.,* 2017), and red deer (*C. elaphus*) (Caslini *et al.,* 2016). Conversely, there may be a temporal delay between stressor impact and population response, such that stressors that initially caused decline were no longer present or were declining during biomarker sampling. These findings point to HCCs’ capacity to be used as a tool to monitor annual changes in HPA axis activity; the temporal associations with population change must be further investigated.

### Hair growth

A major complicating factor for interpreting results in this study was the lack of documentation and understanding of caribou hair phenology. The precise timing of hair growth is rarely well described for wild ungulate species (Nowak *et al.,* 2020), including *Rangifer* (Ashley *et al.,* 2011), whose coat is not part of the commercial textile industry. The general season of guard hair shedding and growth for *Rangifer* is described; however, precise timing and pattern of growth by ecotype are unknown, and the undercoat growth pattern is unknown beyond annual persistence (Cuyler and Oritsland, 2002; Laaksonen and Paulsen, 2015). Guard hair and undercoat HCC of other species are weakly correlated and may have significantly different cortisol concentrations (Macbeth *et al.,* 2010; Dulude-de Broin *et al.,* 2019). Furthermore, the season of guard hair and undercoat growth may not be synchronous in ungulates (Nixon *et al.,* 1991). Although undercoat contamination of our samples is possible, every effort was made to remove the undercoat during the initial sorting process. Nevertheless, improved description of the *Rangifer* hair growth cycle and a comparison of guard hair versus undercoat HCC would improve our understanding of HCC dynamics in *Rangifer spp*.

## Conclusion

Migratory caribou are a keystone species in Canada (Festa-Bianchet *et al.,* 2011; Polfus *et al.,* 2016), with major declines in many herds and populations over the last decade. These declines are variably attributed to cumulative stressors, and the use of HCC as a biomarker of stress holds promise as a tool to monitor chronic stress. Our study used ‘convenience’ samples from harvester-based sampling and capture/collaring operations, and our results highlight the importance of accounting for sample source and location as well as seasonality in study design data interpretation. Accounting for this, we detected HCC variability between herds and trends over time, suggesting this is a useful tool for tracking long-term annual trends of stressors in migratory caribou.

## Funding

This work was supported financially by the W. Garfield Weston Scholarship Foundation/Association of Canadian Universities for Northern Studies, the Northern Scientific Training Program, the Alberta Graduate Excellence Scholarship and the University of Calgary Graduate Scholarship, which were awarded to F.R. The research was supported by grants to S.K. from Polar Knowledge Canada [Grant NST-1718-0015], Environment and Climate Change Canada, Irving Maritime Ship Building—Nunavut Arctic College and NSERC (Natural Sciences and Engineering Council of Canada). Canada North Outfitting and the Governments of Nunavut and the Northwest Territories provided invaluable in-kind support.

## Data availability

The data underlying this article will be shared on reasonable request to the corresponding author.

## Ethics Statement

This research was approved under Animal Care and Use Permit No. AC18–0093, Wildlife Research Permit No. WL2021–053 for Nunavut and Wildlife Research Permit No. WL500877, Wildlife Care Committee Permit No. 2020–008 for the Northwest Territories. Statistical analyses demonstrate that at least 30–40 collars per herd are needed to define seasonal range use necessary for conservation and management (Adamczewski and Boulanger, 2016). Because the animal is being handled for these purposes, the opportunity is presented to gain as much biological information as possible, including the collection of non-invasive health data and samples. Animal handling protocols approved by the Wildlife Care Committee include the collection by trained personnel of hair, blood, feces and mucosal swabs, in addition to demographic and morphometric data.

## Author Contributions

F.R.: Conceptualization, methodology, formal analysis, writing—original draft. X.F-A.: Conceptualization, reviewing, editing. M.P.: Analysis, reviewing, editing. D.W.: Reviewing, editing. G.M.: Methodology, investigation, reviewing, editing. L-M.L.: Resources, reviewing, editing. N.J.: Resources, reviewing, editing. S.K.: Conceptualization, methodology, reviewing, editing.

## Supplementary Material

Web_Material_coad030
