# Supplementary material for: Variation of hair cortisol in two herds of migratory caribou (Rangifer tarandus): implications for health monitoring
Source: Conserv Physiol. 2023 May 22;11(1):coad030. doi: 10.1093/conphys/coad030 (PMC10203588; doi:10.1093/conphys/coad030)
Supplement: Web_Material_coad030 [file web_material_coad030.pdf]

Supporting Information

Variation of Hair Cortisol in Two Herds of Migratory Caribou (*Rangifer tarandus*):

Implications for Health Monitoring

Filip Rakic, Javier Fernández-Aguilar, Mathieu Pruvot, Douglas Whiteside, Gabriella

Mastromonaco, Lisa-Marie Leclerc, Naima Jutha, and Susan Kutz

Table S1. Model estimates of linear-mixed effect model evaluating the effects of body location (neck reference category)

	Coef	SE	DF	t-value	p-value
Intercept	1.67	0.053	51	31.37	<0.001
Rump	-0.236	0.066	51	-3.61	<0.001

Table S2. Summary of linear mixed effect model evaluating the effects of body location

Number of observations	104
Number of groups	52
Intercept variance (between)	0.035
Error/Residual variance (within)	0.111
Marginal R^2	0.09
Conditional R^2	0.31
ICC	0.241

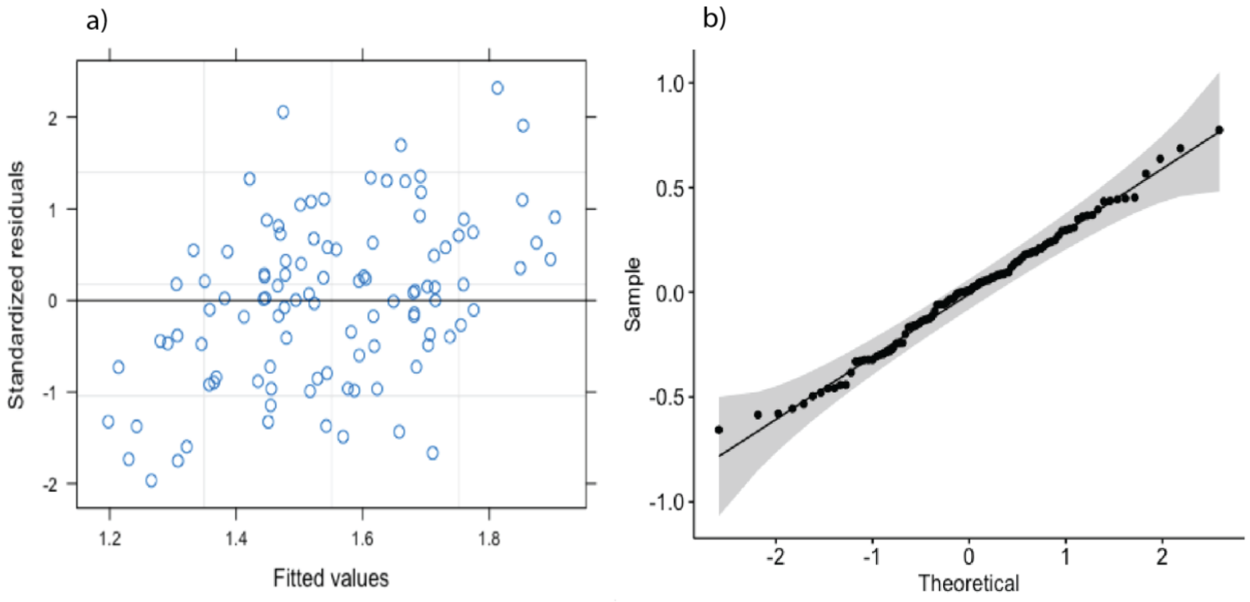

Figure S1. Summary of model fit for body location. A) corresponds to the residuals vs fitted values B) corresponds to a qqplot of the residuals.

Table S3. Summary of top 4 dredged models out of 256 total possible combinations. No top models included an interaction between parameters. Model number (#), included parameters (+ = included, - = excluded), df, AICc,change in AICc (delta), and % model weight into average.

#	Year	Bod Loc	Herd	MI	OI	Month	Sex	Method	df	AICc	delta	weight
1	+	+	+	+	+	+	+	+	12	2246.768	0.00	0.37
2	-	+	+	+	+	+	+	+	11	2247.004	0.235	0.33
3	+	+	+	+	+	+	-	+	11	2248.496	1.728	0.16
4	+	+	+	+	+	+	+	-	11	2248.728	1.959	0.14

Table S4. Summary of averaged model parameters obtained from averaged global model (from Table S3) predicting log caribou hair cortisol concentration, n=407, outliers present. Reference categories in paratheses.

Covariates	Estimate (2.5%: 97.5%)	Std. Error	Z -value	p-value
Year	-0.390 (-0.77: -0.14)	0.191	2.03	0.042
Hair loc-unknown (rump)	-0.67 (-1.79: 1.65)	0.877	0.08	0.903
Hair loc – Neck (rump)	2.921 (1.47: 4.37)	0.734	3.95	<0.001
Hair loc – Shoulder (rump)	-0.691 (-2.91: 1.53)	1.13	0.61	0.527
Herd (BNE)	2.836(1.57: 4.10)	0.644	4.29	<0.001
MI	6.46(-26.05: 38.91)	16.53	0.39	0.697
Month (1-6)	1.01(0.60: 1.40)	0.204	4.89	<0.001
OI	-4.602(-36.79: 27.56)	16.378	0.28	0.739
Sex (Female)	-0.870 (-1.75: 0.049)	0.445	1.94	0.051
Method (Capture)	-1.211 (-2.35: -0.068)	0.581	2.08	0.043

Table S5. Summary of averaged model parameters obtained from averaged global model (from Table S3) predicting log caribou hair cortisol concentration, n=403, outliers removed. Reference categories in paratheses.

Covariates	Estimate (2.5%: 97.5%)	Std. Error	Z -value	p-value
Year	-0.35 (-0.57: -0.12)	0.114	3.04	0.002
Hair loc-unknown (rump)	-0.07 (-0.93: 1.07)	0.512	0.13	0.897
Hair loc – Neck (rump)	2.811 (2.02: 3.60)	0.405	6.93	<0.001
Hair loc – Shoulder (rump)	-0.572 (-1.86: 0.71)	0.657	0.87	0.383
Herd (BNE)	2.990 (2.25: 3.72)	0.377	8.01	<0.001
MI	6.198 (-12.30: 24.70)	9.44	0.66	0.511
Month (1-6)	0.862 (0.63: 1.10)	0.13	7.18	<0.001
OI	-10.782 (-28.95: 7.28)	9.27	1.16	0.244
Sex (Female)	-0.374 (-0.90: 0.15)	0.27	1.39	0.165
Method (Capture)	-1.702 (-2.37: -1.02)	0.344	4.94	<0.001
